# The closterovirus-derived gene expression and RNA interference vectors as tools for research and plant biotechnology

**DOI:** 10.3389/fmicb.2013.00083

**Published:** 2013-04-11

**Authors:** Valerian V. Dolja, Eugene V. Koonin

**Affiliations:** ^1^Department of Botany and Plant Pathology, Oregon State UniversityCorvallis, OR, USA; ^2^Center for Genome Research and Biocomputing, Oregon State UniversityCorvallis, OR, USA; ^3^National Center for Biotechnology Information, National Library of MedicineBethesda, MD, USA

**Keywords:** viral vector, closteroviruses, RNAi, *Beet yellows virus*, *Citrus tristeza virus*, *Grapevine leafroll-associated virus-2*

## Abstract

Important progress in understanding replication, interactions with host plants, and evolution of closteroviruses enabled engineering of several vectors for gene expression and virus-induced gene silencing. Due to the broad host range of closteroviruses, these vectors expanded vector applicability to include important woody plants such as citrus and grapevine. Furthermore, large closterovirus genomes offer genetic capacity and stability unrivaled by other plant viral vectors. These features provided immense opportunities for using closterovirus vectors for the functional genomics studies and pathogen control in economically valuable crops. This review briefly summarizes advances in closterovirus research during the last decade, explores the relationships between virus biology and vector design, and outlines the most promising directions for future application of closterovirus vectors.

## INTRODUCTION

The family *Closteroviridae* has a special place in molecular and evolutionary virology. Together with animal coronaviruses, closteroviruses explore the upper size limit for the RNA-based genomes (Dolja et al., 2006; Gorbalenya et al., 2006). The relatively large genetic capacity of these viruses likely requires higher fidelity of RNA replication than is typical for RNA viruses (Denison et al., 2011), but also allows them to acquire new beneficial genes. On a more practical side, genetic plasticity of closteroviruses makes them attractive vehicles for the delivery and expression of recombinant genes engineered into viral genomes. While generation of coronavirus-based expression vectors seems to be in its infancy, several well-developed closteroviral vectors are available and show strong potential for application in functional genomics and pathogen control (Prokhnevsky et al., 2002; Folimonov et al., 2007; Kurth et al., 2012). Because RNA viruses do not normally integrate their genomes into host chromosomes, utilization of RNA virus vectors provides a useful alternative to transgenic technology helping to bridge the divide between a science-based perspective and the more emotionally charged public perception of genetic engineering and biotechnology.

As is the case for any positive-strand RNA virus, engineering a closterovirus into a vector requires generation of a biologically active cDNA clone. Such full-length genomic clones so far have been reported for *Lettuce infectious yellows virus* (LIYV; Klaassen et al., 1996), *Beet yellows virus* (BYV; Peremyslov et al., 1998), *Citrus tristeza virus* (CTV; Satyanarayana et al., 1999), *Grapevine leafroll-associated virus-2 *(GLRaV-2; Liu et al., 2009), and *Lettuce chlorosis virus* (Mongkolsiriwattana et al., 2011). Although the ability of LIYV to express recombinant proteins has been confirmed (Wang et al., 2009), only BYV, CTV, and GLRaV-2 were developed into gene expression vectors capable of full-fledged systemic infection of the host plants. Furthermore, it was recently shown that the GLRaV-2-derived vector has a capacity to trigger RNA interference (RNAi) that targets host endogenous genes (Kurth et al., 2012), a capacity traditionally called virus-induced gene silencing (VIGS; Baulcombe, 1999).

Admittedly, unlike the *Tobacco mosaic virus* (TMV)-based vectors (Pogue et al., 2002; Gleba et al., 2007), closterovirus vectors are not well suited for rapid mass production of the recombinant proteins. This is the case because of the slower infection cycle and tissue-specific tropism of most closteroviruses whose replication is limited to the phloem (Bar-Joseph et al., 1979). However, closterovirus vectors fill very important niches that are inaccessible to most other plant virus vectors. These niches include fruit-producing specialty crops such as citrus and grapevine, genetic capacity and stability that allow long-term expression of the large recombinant genes, and ability to combine protein expression and VIGS in the same vector. It seems that the scientific base for closterovirus vector application in research and biotechnology is mature. Thus, realization of a strong commercial potential of these vectors depends primarily on the availability of the proper investment.

## GENOME STRUCTURE, REPLICATION, AND EXPRESSION

Currently, the family *Closteroviridae* includes three approved (*Closterovirus, Crinivirus*, and *Ampelovirus*; Karasev, 2000) and one proposed (*Velarivirus*; Al Rwahnih et al., 2012) genera. All closteroviruses share two large, conserved gene modules one of which is responsible for genome replication, whereas the other one functions in genome packaging and intercellular transport (Dolja et al., 2006). The composition of the 3′-proximal genome region varies between and often within the genera. Furthermore, crinivirus genomes are split between two RNAs in contrast to a single genomic RNAs in other genera. Despite the large, up to 19.3 kb size of their genomes, closteroviruses are rank-and-file members of the Alphavirus-like superfamily of the positive-strand RNA viruses (Koonin and Dolja, 1993; Dolja and Koonin, 2011) with capped genomic RNAs that are directly translated to produce an RNA replicase (Karasev et al., 1989; Agranovsky et al., 1994b).

**FIGURE 1:**
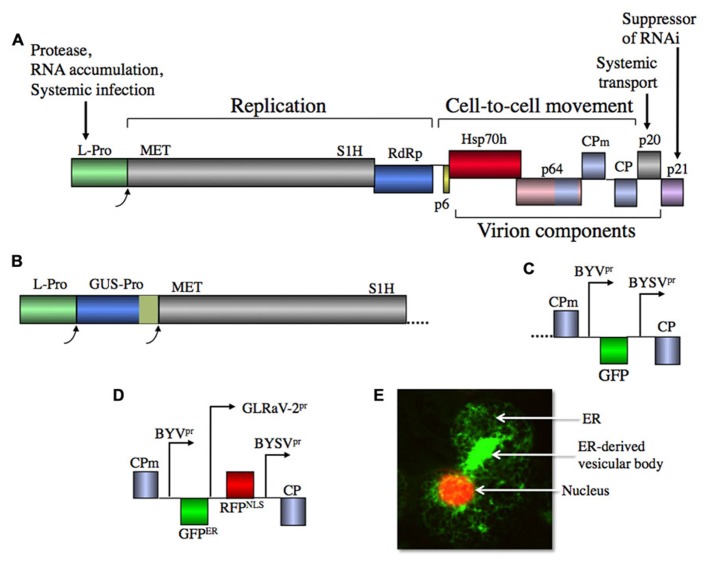
**(A)** Diagram of BYV genome with gene functions shown. L-Pro, papain-like leader protease; MET, methyltransferase (capping enzyme); S1H, superfamily I helicase; RdRp, RNA-dependent RNA polymerase; p6, 6-kDa protein; Hsp70h, Hsp70 homolog; p64, 64-kDa protein; CPm, minor capsid protein; CP, capsid protein; p20, 20-kDa protein; p21, 21-kDa protein. Homologous CP, CPm, and the C-terminal domain of p64 are shown in the same color. **(B)** Proteolytic gene expression cassette including β-glucuronidase ORF (GUS) fused in frame to papain-like, self-cleaving domain of potivirus HC-Pro. **(C)** Recombinant gene expression cassette including native BYV promoter, green fluorescent protein ORF (GFP) and heterologous BYSV promoter. **(D)** Dual expression cassette including heterologous BYSV and GLRaV-2 promoters, ER-targeted GFP and red fluorescent protein containing nuclear localization signal. **(E)** Confocal laser scanning microscopic image of the *N. benthamiana* leaf cell infected with BYV vector that expresses GFP^ER^ and RFP^NLS^. Note the virus-induced, ER-derived, multivesicular body likely containing viral RNA replication complexes.

Because BYV is the prototype member of the family (Bar-Joseph et al., 1979; Dolja, 2003), this and the following sections of the article are focused on BYV with other viruses being evoked as needed. The ~15.5 kb BYV genome encompasses nine open reading frames (ORFs; Agranovsky et al., 1991b, 1994b). The conserved replication gene module includes ORFs 1a and 1b that encode a polyprotein containing methyltransferase (MET), superfamily 1 RNA helicase (S1H), and RNA-dependent RNA polymerase (RdRp; expressed from ORF 1b via +1 translational frameshift) domains (**Figure 1A**). A large central portion of this polyprotein is less conserved, but is functionally important because several alanine-scanning mutations introduced into this region decreased or abolished RNA amplification (D. V. Alzhanova and V. V. Dolja, unpublished data). It seems plausible that this region contributes to the relatively high fidelity of RNA replication required for the reproduction of RNA viruses with the largest genomes, as shown to be the case for coronaviruses (Denison et al., 2011). However, extensive database searches failed to identify significant sequence similarity between the central parts of the closterovirus polyproteins and any other proteins. Moreover, examination of the alignment of the sequences of the closterovirus polyproteins between the MET and the S1H domains failed to identify any conserved motifs resembling those in the catalytic sites of any known nucleases, making it unlikely that enzymes functionally analogous to the proofreading nucleases of coronaviruses lurk in the uncharacterized parts of closterovirus polyproteins (E. V. Koonin, unpublished observations).

The 5′-terminal region of ORF 1a encodes a papain-like leader protease (L-Pro) that is autocatalytically released from the polyprotein; optimal RNA amplification requires functionally intact L-Pro (Agranovsky et al., 1994b; Peremyslov et al., 1998). Interestingly, several closteroviruses including CTV and GLRaV-2 encode a tandem of leader proteases that have evolved via gene duplication and functional divergence (Peng et al., 2001). Although the exact composition of the RNA replication complex of BYV is not known, it has been shown that this complex localizes to endomembrane vesicles that contain ORF 1a and 1b products including L-Pro released from polyprotein via auto-processing (Erokhina et al., 2001; Zinovkin et al., 2003). It was also found that formation of the vesicular complexes occurs via recruitment and reorganization of the endoplasmic reticulum (ER) by the ORF 1a product (e.g., **Figure 1E**), similar to many other positive-strand RNA viruses (den Boon and Ahlquist, 2010).

In addition to the 5′-proximal replication gene module, efficient amplification of BYV requires p21, a 21-kDa protein encoded by the 3′-proximal ORF 8 (Peremyslov et al., 1998). It has been shown that p21 is a strong suppressor of RNAi that non-specifically binds and sequesters double-stranded form of the small interfering RNAs (siRNAs), and micro RNAs (miRNAs; Reed et al., 2003; Chapman et al., 2004). The homologs of p21 are conserved throughout the genus *Closterovirus* (Chiba et al., 2006), but not in more distantly related viruses; however, RNAi suppressors unrelated to p21 were identified throughout the family (Lu et al., 2004; Kreuze et al., 2005).

Typical of the Alphavirus-like superfamily, expression of the ORFs downstream of the replication gene module occurs via generation of the positive-strand subgenomic RNAs (sgRNAs). These sgRNAs are collinear and 3′-coterminal with the genome, and are functionally monocistronic, expressing only the 5′-terminal ORF. The BYV genome transcription produces seven sgRNAs that have minus-strand counterparts (Dolja et al., 1990). The proteins encoded by the ORFs 2–7 are involved in virion assembly and virus transport within plants (see below).

The transcription start site mapping for the five BYV sgRNAs revealed a somewhat lax sequence conservation pattern in the upstream regions presumed to form sgRNA promoters (Agranovsky et al., 1994a; Peremyslov and Dolja, 2002; Vitushkina et al., 2007). It was also shown that the sgRNA synthesis in BYV is regulated both quantitatively and temporally by several early and late promoters (Hagiwara et al., 1999). The promoter controlling production of the major capsid protein (CP) directs gene expression early in the replication cycle and to the highest level.

More extensive analysis of the genome transcription in CTV produced a complex picture suggesting that each sgRNA promoter can also act as a terminator. As a result, each “normal” positive-strand sgRNA has not only a minus-strand, but also a plus-strand counterpart that expands to the 5′-terminus of genome (Gowda et al., 2001). Furthermore, additional, ~800 nts-long, plus-strand, 5′-coterminal sgRNAs were also described (Che et al., 2001). The exact mechanisms whereby such a complex population of sgRNAs is produced are difficult to interpret in functional terms or to fit into any of the three major expression strategies employed by other positive-strand RNA viruses, namely: (i) internal initiation on a minus-strand; (ii) premature termination of the minus-strand synthesis followed by use of this strand to produce plus-strands; (iii) common leader-initiated, discontinuous synthesis of minus-strand templates for plus-strand sgRNAs typical of coronaviruses (Miller and Koev, 2000; Pasternak et al., 2006; Sztuba-Solińska et al., 2011). The unusually complex pattern of expression in CTV is exacerbated by the promiscuous initiation of the 3′- and 5′-coterminal sgRNAs that appear to use distinct controlling elements within the same promoter region (Ayllón et al., 2003, 2004).

## VIRION MORPHOLOGY, VIRUS TRANSPORT AND TRANSMISSION

The flexuous filamentous virions of closteroviruses are the longest currently known, reaching the length of ~2,000 nm; these virions are built of CPs that are helically arranged around genomic RNA. The overall morphology of the closterovirus capsids is similar to that of the other filamentous viruses in the families α-, β-, and γ-*Flexiviridae* (Martelli et al., 2007), and *Potyviridae* (Adams et al., 2012), all of which encode homologous CPs (Dolja et al., 1991). However, closteroviruses are distinguished by a remarkable structure that caps one end of the virion and was unwittingly called the “tail” by its discoverers (Agranovsky et al., 1995). Subsequently, it was shown that this ~100 nm-long structure encapsidates the 5′-terminal ~650 nts (4%) of the genome and accordingly rather represents a “snout” that measures ~8 nm in diameter compared to the 12 nm virion “body” (Peremyslov et al., 2004). Nevertheless, to avoid confusion, we will continue to use the term “tail” throughout the article. The main building block of the virion tail is the minor CP (CPm; Agranovsky et al., 1995; Satyanarayana et al., 2004) that is a divergent duplicate of the major CP which forms the long virion body (Boyko et al., 1992).

In addition to CP and CPm, the virions of closteroviruses contain at least two more structural proteins. The third one is a ~60-kDa protein (p64 in BYV) whose C-terminal domain is yet another divergent duplicate of the CP (**Figure 1A**; Tian et al., 1999; Satyanarayana et al., 2000; Napuli et al., 2003). The fourth and most unusual virion protein is a homolog of cellular molecular chaperones of the heat shock protein 70 (Hsp70) family, Hsp70 homolog (Hsp70h; Agranovsky et al., 1991a). The viral Hsp70h, however, is a “misbehaving chaperone” that does not leave the scene following successful matchmaking. It was shown that Hsp70h is an integral virion component (Tian et al., 1999; Napuli et al., 2000) that is required for proper virion tail assembly (Satyanarayana et al., 2000; Alzhanova et al., 2001, 2007). Although CPm alone can initiate virion assembly, coordinated incorporation of CPm, the ~60-kDa protein and Hsp70h is required for efficient assembly of the tails of the correct length (Satyanarayana et al., 2000, 2004; Alzhanova et al., 2001, 2007; Napuli et al., 2003).

As was shown for BYV, the complexity of the closterovirus particles does not stop at four structural proteins and includes a fifth, ~20-kDa protein (p20) that incorporates into virions via interaction with Hsp70h (Prokhnevsky et al., 2002). Moreover, analysis of the BYV tail morphology and composition indicated that p20 most likely forms the pointed tip segment of the three-segment tail, with two other segments assembled of CPm, the ~60-kDa protein and Hsp70h (Peremyslov et al., 2004).

The cell-to-cell movement of closteroviruses turned out to be a no less engaging story than that of virion assembly. The closteroviruses possess a conserved dedicated movement protein of ~6-kDa (p6 in BYV) that is targeted to ER via its N-terminal transmembrane domain (Alzhanova et al., 2000; Peremyslov et al., 2004). However, each of the CP, CPm, Hsp70h, and p64 is also indispensable for the cell-to-cell movement of BYV (Peremyslov et al., 1999; Alzhanova et al., 2000; Napuli et al., 2003). Taken together, tight functional coupling of the virion assembly and cell-to-cell movement (Alzhanova et al., 2001) and an ability of Hsp70h to target plasmodesmata in association with microfilaments and class VIII myosin motors (Medina et al., 1999; Prokhnevsky et al., 2005; Avisar et al., 2008) prompted a hypothesis that the virion tail is a movement device (Dolja, 2003; Dolja et al., 2006). The encapsidation of 5′-terminal region of viral genome by the tail is compatible with this hypothesis implying that the Hsp70h-containing tails guide viral genomes to and through plasmodesmata to allow directional transport and translation of viral genomes entering the neighboring cell.

Another twist of the “tail as a transport device” concept was the finding that the virion tip component p20 is required for the long-distance transport of BYV through the phloem (Prokhnevsky et al., 2002). Because BYV p20 shows little if any sequence similarity to proteins in other closteroviruses, it is not clear if these viruses also encode the analogous long-distance transport factors. In contrast, the leader proteases that also are implicated in the long-distance transport of BYV and GLRaV-2 (Peng et al., 2002, 2003; Liu et al., 2009), are conserved throughout the family (Peng et al., 2001).

The semi-persistent vector transmission of closteroviruses relies on three distinct taxa of insects, aphids (*Closterovirus*), mealybugs (*Ampelovirus*), and whiteflies (*Crinivirus*; Karasev, 2000; Ng and Falk, 2006). It is not known which viral proteins mediate aphid- or mealybug-dependent transmission of the viruses in two former genera. For criniviruses, there is strong experimental support for the critical role of CPm in transmission (Tian et al., 1999; Stewart et al., 2010; Chen et al., 2011) suggesting that CPm and/or other tail components are the transmission determinants in other closteroviruses as well.

## CLOSTEROVIRUS BIOLOGY AND VECTOR DESIGN

It should be emphasized that the infection cycle of closteroviruses is relatively slow, with BYV moving from cell-to-cell at a rate of ~1 cell per day (Peremyslov et al., 1999), not every 2–4 h as is the case for TMV (Kawakami et al., 2004). Similarly, the onset of BYV systemic infection occurs at 2–3 weeks post inoculation (Prokhnevsky et al., 2002) compared, for instance, to 3 days for *Tobacco etch potyvirus* (Dolja et al., 1992). The pace of systemic infection for closteroviruses that infect woody plants, such as CTV or GLRaV-2, is even slower, reaching 1 month or longer (Folimonov et al., 2007; Kurth et al., 2012). Furthermore, whereas BYV is able to infect leaf mesophyll and epidermal cells, most of the other closteroviruses are strictly limited to phloem where they are acquired by vectoring insects and deposited by viruliferous insects at the beginning of each infection cycle (Ng and Falk, 2006). These aspects of the virus biology have major impact on development of the proper inoculation techniques for closterovirus-derived gene vectors. Similarly, virus biology has to be taken into account when the utility of the viral vectors or safety measures preventing vector escape during propagation are considered.

The genome organization and molecular biology of closteroviruses are also of paramount importance for the vector design. The recombinant sequence could be either spliced into the virus vector genome, or used to replace part of it. Due to the “wall-to-wall” organization of viral genomes, the replacement strategy almost inevitably results in a loss-of-function phenotype. For instance, there are no non-essential genes in BYV (Dolja, 2003); replacement of any gene will result in a loss of replication or systemic infectivity. In contrast, the larger CTV genome contains genes that are required for infection of some citrus species but not others (Tatineni et al., 2011). These genes, albeit expressed to low levels, are potential replacement targets for vector design.

The mechanisms whereby Alphavirus-like viruses including closteroviruses express their proteins involve polyprotein processing by proteases and expression of sgRNAs (Dolja and Carrington, 1992; Miller and Koev, 2000). Because the closterovirus leader proteases appear to cleave only *in cis*, the proteolytic expression of recombinant protein can be ensured either by fusing the protein to L-Pro, or by inserting a new proteolytic cassette, similar to original designs of the potyviral vectors (**Figure 1B**; Dolja et al., 1992; Carrington et al., 1993).

Engineering of an autonomous expression cassette controlled by an additional sgRNA promoter is the preferable approach to closterovirus vector design. This approach allows one to choose sgRNA promoter of desired strength and to place the cassette into an optimal genomic location. The source of the additional promoter, however, is of paramount importance. If the homologous promoter is duplicated, an added expression cassette can be readily eliminated via homologous recombination. An elegant solution to this problem, utilization of a heterologous sgRNA promoter from a related virus, was advanced originally for a TMV vector (Donson et al., 1991; Dawson, 2011). Because the heterologous promoter has a distinct nucleotide sequence, homologous recombination is effectively eliminated and vector stability increases. Obviously, the activity of the heterologous promoter could be lower than it is in the natural background, so picking the right promoter is a matter of trial and error.

Two other aspects of closterovirus biology important for the vector design are the virion morphology and the inherent large size of the genomes. Unlike icosahedral virions with their limited genome packaging capacity, elongated virions do not set an upper limit to the size of the expression cassette. It also stands to reason that due to their large genomes, closterovirus vectors are better suited for accommodating recombinant expression cassettes than more rigidly organized genomes of smaller RNA viruses such as TMV.

The utility of closteroviruses as VIGS vectors seemed uncertain from general considerations. Unlike well established *Potato virus X *(PVX)- or *Tobacco rattle virus *(TRV)-based VIGS vectors (Baulcombe, 1999; Bachan and Dinesh-Kumar, 2012), closteroviruses encode RNAi suppressors that are among the strongest characterized so far (Reed et al., 2003; Lu et al., 2004; Chiba et al., 2006). However, this did not preclude development of the powerful VIGS vector from at least one closterovirus (see the GLRaV-2 section below).

## BYV-DERIVED VECTORS

Development of the first BYV vectors rapidly followed the generation of the biologically active cDNA clone (Peremyslov et al., 1998; Hagiwara et al., 1999). In these vectors, the β-glucuronidase (GUS) reporter was fused to three different BYV proteins. In addition, a minireplicon that produced only the replication-associated proteins and a free GUS reporter controlled by the CP sgRNA promoter was engineered. Interestingly, accumulation of GUS sgRNA expressed by this minireplicon was ~3.5-fold higher than that of CP sgRNA in the wild type genome background (Hagiwara et al., 1999). In general, relocation of the sgRNA promoter closer to the 3′-end of the closterovirus genome increases its expression levels, an important consideration for optimal vector design.

A more advanced BYV vector capable of expressing recombinant protein from an autonomous cassette has become a prototype for the subsequent designs of vectors based on other closteroviruses (Peremyslov et al., 1999). In this vector, the recombinant ORF encoding green fluorescent protein (GFP) was inserted downstream from the native CP sgRNA promoter, whereas a heterologous CP promoter derived from a closely related *Beet yellow stunt virus* (BYSV; Karasev et al., 1996) was used to express the BYV CP (**Figure 1C**). The infectious RNA transcripts for plant inoculation were obtained *in vitro* using bacteriophage SP6 RNA polymerase and plasmid linearized near the 3′-end of the viral cDNA (Peremyslov and Dolja, 2007). This vector was useful for mechanical inoculation of a highly susceptible BYV local lesion host *Claytonia perfoliata*, whereas systemic infection of a convenient systemic host, *Nicotiana benthamiana*, using RNA transcripts was inefficient.

The next generation of BYV vectors suited for systemic infection of a host plant involved replacement of the SP6 promoter with the 35S *Cauliflower mosaic virus* promoter active in plants, and insertion of a ribozyme downstream from the viral cDNA to ensure proper processing of the resulting viral transcript (Prokhnevsky et al., 2002). This design, originally proposed by Leiser et al. (1992) allowed the use of *Agrobacterium* for efficient delivery of viral cDNA to plant cells mediated by T-DNA-containing binary vectors. The resulting agroinoculation procedure, further improved by vacuum infiltration of the bacterial suspension (Marillonnet et al., 2005), remains the method of choice for introducing RNA viral vectors back to plants.

In general, transient expression of recombinant genes via *Agrobacterium*-mediated transformation is highly efficient because the leaf infiltration procedure delivers large numbers of gene transfer-competent bacteria per each plant cell. In *N. benthamiana*, this technique results in high-level production of a recombinant protein in virtually all exposed cells. Surprisingly, when agroinfiltration is used to deliver the viral vector, only very few cells become infected (Marillonnet et al., 2005; Chiba et al., 2006). Two strategies were proposed to improve the cell infection rate following agroinoculation: (i) labor-extensive vector modifications aimed at suppression of the accidental splicing of the viral transcripts in the transfected plant nuclei (Marillonnet et al., 2005) and (ii) co-expression of the strong RNAi suppressors during agroinoculation (Chiba et al., 2006). Each strategy resulted in a drastic, three-orders of magnitude increase of the infection rate. Interestingly, when a combination of both strategies was attempted for GLRaV-2-derived vectors, it was found that RNAi suppression overrides the need for splicing modification. Thus, the simple use of an RNAi suppressor to supplement agroinoculation appears to be the method of choice for improving vector infectivity.

Further elaboration of the BYV vectors involved engineering of tandem expression cassettes. In a dual expression vector, the GLRaV-2-derived CP sgRNA promoter was inserted upstream from BYSV promoter to allow simultaneous production of the monomeric red fluorescent protein (mRFP) targeted to nucleus and the ER-targeted GFP (**Figures 1D,E**). An alternative vector design included a proteolytic expression cassette introduced downstream from L-Pro; this cassette encompassed a fusion of GUS to the proteolytic domain of the potyvirus helper component-protease (**Figure 1B**). Thus, BYV was proven to provide a facile platform for various vector designs showing genetic plasticity so far unmatched by other plant virus-derived vectors.

At the time of their generation, the genetic stability of the BYV vectors was greater than that of vectors based on other plant viruses. The potyvirus-based vectors could maintain reporter expression for up to 1 month when propagated in the same plant (Dolja et al., 1993), whereas BYV vectors were at least twice as stable (V. V. Peremyslov and V. V. Dolja, unpublished data). For comparison, the PVX-based vectors did not maintain reporter expression even within one cycle of systemic infection lasting around 2 weeks (Chapman et al., 1992). Thus, reporter-expressing BYV vectors provided a facile experimental model for the identification of the genes involved in virus replication, assembly, cell-to-cell movement and systemic transport (Dolja et al., 2006). Regrettably, the utility of these vectors for gene expression or VIGS in the economically relevant BYV hosts such as sugar beet or spinach has not been so far assessed.

## CTV-DERIVED VECTORS

The generation of the full-length, biologically active cDNA clone of CTV was more challenging than it was for BYV. This was mainly because CTV genome is larger than the BYV genome and because unlike BYV, CTV does not normally infect herbaceous plant species. Due to the low infectivity of full-length transcripts of the CTV cDNA in protoplasts, most of the initial experimentation was performed with minireplicons (Satyanarayana et al., 1999). To overcome this limitation, a laborious procedure of cyclic virion transfer in protoplasts initially transfected with RNA transcripts was developed (Satyanarayana et al., 2000). This procedure was also adapted for slash-inoculation of citrus trees with virions propagated in protoplasts (Satyanarayana et al., 2001).

The later development of CTV-based vectors expressing the GFP reporter produced the best results with the design mirroring that of the BYV vector; a short variant of the BYV CP sgRNA promoter was used to drive GFP expression (Peremyslov et al., 1999; Folimonov et al., 2007). Most remarkably, the genetic stability of CTV vector in citrus proved to be much higher than the stability of the BYV vector in *N. benthamiana*. Although gradual loss of the expression cassette occurred in some of the vector-infected trees, many trees maintained GFP expression for over 2 years (Folimonov et al., 2007) and some even longer, up to 7 years (Dawson, 2011).

Recently, an agroinoculation procedure to introduce CTV to *N. benthamiana* has been developed (Ambrós et al., 2011). Surprisingly, a CTV vector launched by an *Agrobacterium* was not only systemically infectious in this presumed non-host plant, but was able to exit the phloem to which it is strictly limited in the citrus hosts. Similar to BYV, the efficiency of agroinoculation was increased by addition of RNAi suppressors (Chiba et al., 2006; Ambrós et al., 2011). Although an agroinoculation technique to infect citrus is not yet available, the ability to propagate CTV-derived gene expression vectors in *N. benthamiana* rather than in protoplasts will facilitate investigation of CTV gene functions. It will be interesting to determine if genetic stability of the CTV vectors in a herbaceous host matches that in citrus.

The CTV vector has a significant potential not only for the research on the gene functions or virus population dynamics in the infected citrus (Folimonova et al., 2010; Tatineni et al., 2011), but also in the development of pathogen-resistant citrus trees (Dawson, 2011). However, this potential might be jeopardized by the concerns due to the CTV transmission by its natural insect vectors, aphids. Even if the CTV transmission factors are identified and disabled without affecting vector infectivity, transmissibility of such disarmed vectors could be restored via recombination with the wild CTV isolates that are ubiquitous in the agricultural settings.

## GLRaV-2-DERIVED VECTORS

The latest addition to the assortment of closterovirus gene vectors were the vectors based on the GLRaV-2 (Liu et al., 2009; Kurth et al., 2012). The first generation of these vectors was used to dissect functions of the two leader proteinases (L1 and L2) in the experimental host *N. benthamiana* and to determine that both of them provided varying contributions to the establishment and systemic spread of virus infection (Liu et al., 2009). Unexpectedly, the significance of these proteases was much greater for the infection of grapevine leaf cells compared to that in *N. benthamiana*, attesting to the host-specific roles of L1 and L2 in virus infection. These vectors, however, failed to systemically infect grapevine; it took us several years of sustained effort to identify the culprit and to find a solution of this problem.

There turned out to be two major impediments to the development of a virus vector for grapevine. Unlike CTV whose ability to infect citrus trees upon slash-inoculation was established long ago (Garnsey et al., 1977), to the best of our knowledge, there have been no reports of successful mechanical inoculation of grapevine with any virus. Thus, we had to rely on agroinoculation without knowing if this technique was suitable for virus launching to phloem tissue where it naturally reproduces. Paradoxically, GLRaV-2 can be mechanically transmitted to *N. benthamiana* (Goszczynski et al., 1996), seemingly a blessing that turned to be a curse.

The initial full-length cDNA clone was obtained using *N. benthamiana*-propagated GLRaV-2; this clone was readily launched to this host plant via agroinoculation, and exhibited primarily phloem-limited systemic distribution as one would expect (Liu et al., 2009). To overcome the lack of systemic infectivity in grapevine, we tested a number of potential solutions: (i) addition of homologous and heterologous RNAi suppressors (Chiba et al., 2006); (ii) improving vector infectivity via eliminating potential sites of aberrant splicing and adding plant-specific introns (Marillonnet et al., 2005); (iii) a combination of (i) and (ii); (iv) all possible means of mechanical inoculation from rubbing to pinching to slashing to bombarding with microparticles to vacuum infiltrating; (v) testing agroinoculation of grapevine roots (Muruganantham et al., 2009), young seedlings, or micropropagated plantlets; and, (vi) screening for the optimal *A. tumefaciens* and *A. vitis* strains.

When all these possibilities were exhausted, we reasoned that there must have been a problem with the cDNA clone itself. Because the viral RNA genomes are prone to rapid mutation accumulation and thus rapidly evolve to adapt to a new host (Roossinck and Schneider, 2006), propagation of GLRaV-2 in *N. benthamiana* could result in the loss of infectivity in grapevine. Accordingly, we embarked on a wholesale reassembly of our vector with cDNA fragments derived from GLRaV-2-infected grapevine. It is important to emphasize that only cDNAs corresponding to a consensus sequence of an isolate that were likely to represent the dominant infectious variant were used. Compared to the complete consensus sequence of the grapevine isolate, the original vector had 75 point mutations some or all of which could contribute to the loss of vector infectivity in grapevine.

**FIGURE 2:**
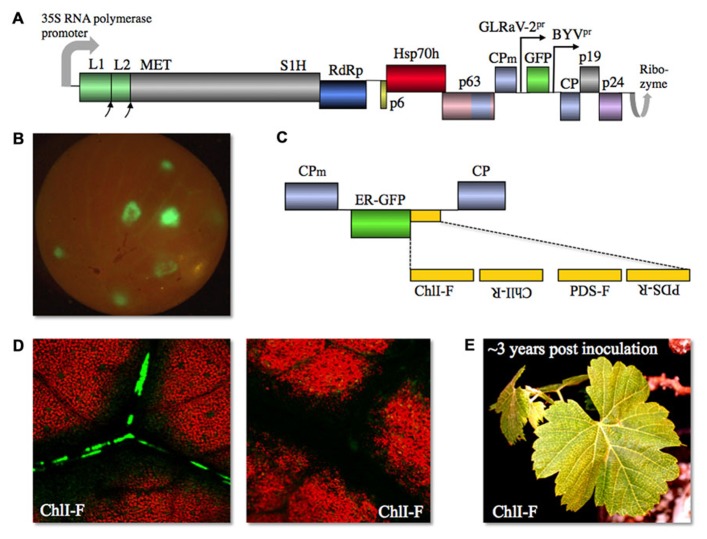
**(A)** Diagram of GLRaV-2-based gene expression vector vLR2-GFP with gene functions shown. L1 and L2, papain-like leader proteases 1 and 2; p19, 19-kDa protein; p24, 24-kDa protein; other designations as in **Figure 1A**. **(B)** vLR2-GFP-mediated GFP expression in grapevine (*Vitis vinifera*) berry. **(C)** Diagram of vLR2-based dual vector in which four variants of ChlI- or PDS-derived sequences in forward (F) or reverse (R) orientation were inserted downstream of ER-GFP ORF. **(D)** Spread of the dark, chlorophyll-less areas starts around the *V. vinifera* leaf cells infected with vLR2-ER-GFP-ChlI-F (green) as shown in the left panel, and later expands along the veins into the areas that contain no virus-infected cells (right panel). **(E)** Grapevine leaves showing chlorotic symptoms due to the RNAi targeting ChlI ~3 years post inoculation with vLR2-ER-GFP-ChlI-F.

Using the optimized procedure of vacuum agroinfiltration of the whole, micropropagated plantlets, we obtained consistent systemic infection of several grapevine varieties with this second generation, “grapevinized,” GFP-expressing, GLRaV-2 vector dubbed vLR2-GFP (**Figure 2A**; Kurth et al., 2012). It was found that the vector-infected cells appeared first in the stem bark phloem and then colonize leaf petioles, midrib, and smaller veins between 3 and 6 weeks post inoculation. After several months of propagation, vLR2-GFP started to accumulate in the root phloem. Conspicuously, when the berries were formed, infection invaded some of them spreading initially through the phloem bundles and then exiting into mesocarp cells (**Figure 2B**; Kurth et al., 2012). Once established, the vector infection can be readily transmitted by grafting to apparently any variety of table or wine grapes. Similar to CTV vectors, vLR2-GFP is genetically highly stable: only some of the infected plants exhibited deterioration of the insert after 1 year-long propagation in grapevine (Kurth et al., 2012).

The most unexpected and important ability of vLR2 was to elicit powerful systemic VIGS despite the fact that GLRaV-2 encodes a strong RNAi suppressor p24 (Chiba et al., 2006). This ability was validated using two endogenous grapevine genes involved in chlorophyll metabolism as VIGS targets. These genes were phytoene desaturase (PDS) and subunit I of magnesium-protoporphyrin IX chelatase (ChlI); nucleotide sequences derived from each of the corresponding ORFs were inserted into vLR2-GFP in the positive or negative orientations either downstream of the GFP ORF or as replacement of the GFP ORF (**Figure 2C**). Each of these vector variants was inoculated to grapevine and each induced a strong VIGS response manifested as leaf discoloration due to chlorophyll loss (Kurth et al., 2012). The chlorophyll-less cells appeared first nearby the virus-infected cells and then VIGS spread along the veins systemically and into leaf mesophyll and epidermis (**Figure 2D**) as is typical for VIGS elicited by other vectors (Baulcombe, 1999). The PDS and ChlI VIGS phenotypes proved to be long-lasting; they were maintained in most of the infected plants for over 1.5 years (Kurth et al., 2012). Furthermore, some of the plants exhibited the VIGS phenotype after nearly 3 years of propagation (**Figure 2E**).

Thus, the vLR2 vector has a dual capacity for recombinant gene expression in the phloem and systemic VIGS targeting endogenous host genes or, potentially, genes of pathogens or pests that parasitize the grapevine. Thus, this vector provides powerful tools for functional genomics and pathogen control in grapevine. Because GLRaV-2 is known to be transmitted only by grafting, potential genetically modified organism (GMO) safety concerns are greatly reduced promoting commercial application of this vector.

## CURRENT AND FUTURE CHALLENGES

Over a decade of research into generation of the closterovirus-derived gene vectors taught us several valuable lessons. One of these is the paramount significance of the meticulous reconstruction of the viral cDNA representing the genome variant that is the most fit within the virus population in a systemically infected natural host plant. Another lesson is the importance of development of the optimal plant inoculation technique. Although agroinfiltration remains by far the most efficient and broadly applicable among these techniques, it needs to be tailored for each virus–host combination, particularly for the woody hosts.

It is unlikely that closterovirus vectors will ever over-compete TMV or TRV for the tasks of facile protein production or VIGS in common herbaceous plants such as tobacco. However, neither TMV nor TRV are capable of infecting citrus or grapevine, or maintaining the recombinant gene expression cassette for years. Therefore, closterovirus vectors provide unique and extremely valuable tools for citrus and grapevine biotechnology. The VIGS capability of vLR2 is an excellent example of the power of closterovirus vectors. This vector is immediately applicable to the functional genomics of grapevine whose complete genome has been sequenced (Jallion et al., 2007; Velasco et al., 2007). Compared to other tools of functional genomics such as plant transformation, VIGS is much less time- and labor-consuming and thus is the method of choice for mapping the grapevine genes that control pathogen resistance, berry physiology, or nutrient content. It seems all but certain that the use of vLR2 will greatly facilitate the quest for more environmentally friendly and sustainable viticulture, as well as for the grapes that are more nutritious, beneficial for health, or make for better wines.

Another potential application of closterovirus vectors is development of RNAi-mediated resistance to the RNAi-susceptible pathogens such as viruses and fungi, or pests, such as insects or nematodes. For vLR2-based VIGS vectors, the obvious targets are mildew-causing fungi, phylloxera, mealybugs, and glassy-winged sharpshooters. It should be emphasized that mealybugs and sharpshooters are not only pests, but also vectors that transmit viruses causing leafroll disease and bacterium *Xylella fastidiosa* causing Pierce’s disease, respectively. Obviously, to be useful for disease protection, viral vectors themselves need to exhibit as low pathogenicity as possible. The GLRaV-2 infection causes relatively mild disease facilitating the use of vLR2 vectors as a “lesser evil” to fight devastating diseases, e.g., GLRaV-3 infection or Pierce’s disease. Perhaps, an even better virus vector for grapevine could be generated using GLRaV-7 that causes symptomless infections in many grape varieties (Al Rwahnih et al., 2012).

A major strength of closterovirus vectors is their exceptional genomic stability unmatched by other plant virus vectors. The causes of this stability, however, remain enigmatic. One possible explanation is the viral population dynamics related to strict tissue tropism of most closteroviruses including GLRaV-2 and CTV. An initial phase of infection by a phloem-limited virus could involve massive loading to sieve elements from one or a few initially inoculated companion of phloem parenchyma cells. Such loading would avoid multiple genetic bottlenecks associated with the cell-to-cell movement of other viruses that traverse many epidermal and mesophyll cells before reaching the phloem. Accordingly, the recombinant cassette-possessing vector that initiates the infection faces less competition from the more fit deletion variants that lose the cassette. Another explanation is higher RNA replication fidelity provided by the unusually large closterovirus replication polyprotein; elucidation of the molecular mechanisms behind this high replication fidelity remains an interesting challenge for further work on closteroviruses. Virus population dynamics and replication fidelity could act in synergy resulting in the sustained years-long expression of the recombinant proteins or RNAi triggers.

We would like to conclude this brief overview on a somewhat personal note. Since we have started to investigate closteroviruses over two decades ago, we continuously enjoyed finding many surprising features that distinguish these viruses from their smaller and less sophisticated kin. These features included the first virus-encoded molecular chaperone that turned to be a dispatcher of virion assembly and virus transport, triplication of the CP gene that provided building blocks for the formation of unusual polar virions, discovery of several diverse RNAi suppressors, extreme versatility and stability of closteroviral gene vectors and more. However, several important problems including the exact function of the unique domains in the RNA replicase, mechanisms of insect transmission, cooperation between Hsp70h, myosins, plasmodesmata, and ER-targeted movement protein that empowers cell-to-cell movement, molecular functions of the leader proteases or AlkB domain present in some closteroviruses, remain unsolved. It is our hope that the available advanced models including BYV, CTV, GLRaV-2, and LIYV will be used to address these and other outstanding problems of molecular plant virology.

## Conflict of Interest Statement

The Research in Valerian V. Dolja lab is supported in part by Vinoculate, Inc. (Soledad, CA).
